# MoS_2_ with Organic Fragment - a New Hybrid Material for Laser Writing

**DOI:** 10.1038/s41598-019-44085-7

**Published:** 2019-05-24

**Authors:** Arunas Jagminas, Romualdas Trusovas, Carla Bittencourt, Marija Kurtinaitienė, Vidas Pakštas, Damien Cossement, Gintaras Valušis

**Affiliations:** 1 State Research Institute Center for Physical Sciences and Technology, Saulėtekio av. 3, LT-10257 Vilnius, Lithuania; 2 Chemie des Interactions Plasma-Surface, University of Mons, Place du Parc 22, 7000 Mons, Belgium; 3 Materia Nova Research Center, Avenue Copernic, 3, 7000 Mons, Belgium

**Keywords:** Chemical synthesis, Materials science, Optical materials and structures

## Abstract

New nanostructured metasurfaces capable change the composition and physical properties upon pulse laser excitation recently received a marked attention for nanophotonic technologies. In this study, well adherent to the metal substrate and significantly thicker nanoplatelet-shaped MoS_2_-based arrays were synthesized by one pot hydrothermal way *via* addition of ethanolamine in the synthesis solution containing ammonium heptamolybdate and thiourea. It was shown that the lightening of this material with green light *ns*-laser pulses at a suitable fluencies results in the detachment of organic species and compositional transformations to significantly pure MoS_2_ material. For characterization the synthesized products scanning electron microscopy (SEM), glancing angle X-ray diffraction (GA-XRD), diffuse reflection, Raman, and time-of-flight secondary ion mass spectrometry (ToF-SIMS) methods before and following green light picosecond laser pulse illumination were applied. We envisaged that these films can be successfully used as metamaterial for laser writing.

## Introduction

In the modern nanophotonic hybrid systems composed of a resonant metal and dielectric counterpart, their design and properties can be effectively tuned by light^1,2^. The effect of laser painting onto the surface of photonic materials resulting in their color changes is well known^3^. There are also a number of metamaterials capable to change the composition and physical properties upon the powerful *fs*-laser light heating^4,5^. A typical insulator-to-metal transition in vanadium dioxide, driven by an ultra-fast laser pulse illumination, currently is widely used in photonic devices^6^. The transition between crystalline and amorphous phases upon the *fs*-laser pulse treatment has been also reported for silicon nanospheres^7^ and Ge-Sb-Te (GST) nanostructured alloys^8,9^. For example, in nanophotonic GST amorphous structures, the laser-induced crystallization proceed by a single-pulse laser excitation at a wavelength of 660 nm with 50 ns pulses at a peak intensity of about 0.25 mW cm^−2^ whereas reverse transition^10^ takes place upon excitation with 100 ns pulses at a fluency around 1.0 mJ cm^−2^. Laser-induced chemical vapor deposition and growth of Ge nanowire on the locally illuminated Au NP due to resonant light absorption by Au NP and Ge supersaturation is a sample of the typical non-reversible process induced by the local *fs*-laser pulse treatment^11^. In this way, the optical properties of Ge nanowire can simple be tuned by changing the laser power. The reshaping of gold nanowires, comprised plasmonic *meta*-surfaces, using *fs*-laser pulses at the fluency high enough to melt them and diffuse of gold atoms from the tip to the center part of the rods has been reported in several papers^12–14^. Besides, at the highest fs-pulse lased intensities, it is possible to achieve material removal taken place through the local melting, high pressure generation and stress-induced deformation^15^. However, the information about the materials capable change their composition and physical properties upon the green or red light ns-pumping is scarce because these hybrid *meta* systems combining both plasmonic and magnetic resonance features required multistep fabrication processing.

The fabrication of 2D nanocomposites assembled from the transition metal dichalcogenides, such as MoSe_2_, WS, MoSe_2_, and graphene possessing ultrafast and nonlinear optical properties associated with their ultra-thin 2D-structure and strong light-matter intercalation^16–18^ motivated us to design the new and cost-effective composite 2D materials with similar properties. Our strategy was based on the variety of two-dimensional (2D) molybdenum disulfide (MoS_2_) crystals with characteristic –S-Mo-S- few-layered structure exhibiting three typical phases, namely 2H, 1T, and 3R recently holding tremendous interest because of their intriguing properties and potential applications^19,20^. A trigonal prismatic arrangement of Mo atoms in 2H- and 3R-MoS_2_ induces them semiconducting properties^21^ whereas the Mo atoms with octahedral prismatic coordination in the 1T-MoS_2_ provided metallic features^22^. Upon alkali metal ion intercalations^23^, as well as O_2_ and Ar molecules treatment^24^, 2H-MoS_2_ transforms to 1T phase. However, phase transformation in exfoliated and gas-treated MoS_2_ of the single and few-layered sheets usually is not complete and leads to formation of the layers containing both 2H- and 1T-MoS_2_ fractions^25^. Note that advantage of 1T and more stable disordered 1T (1T’) phases still remains a topic of debate^26^. To increase the stability and catalytic efficiency, intercalation of ammonium^27^ and methylammonium^28^ *via* hydrothermal synthesis approach have been proposed.

Recently, we have found that the defects in the MoS_2_ nanoplatelets, fabricated by a wet processing way, can significantly be decreased *via* the green light excitation^29^. Upon this fact, in this study we succeed in the design of novel hybrid-type MoS_2_ films with the entrapped ethanolamine fragments which are sensitive to the green light *ns*-pumping. In this way, the few times thicker, nanoplatelet-shaped film composed of semiconducting 2H-MoS_2_ and semi-metallic 1T-MoS_2_ phases heterostructured with attached organic fragments demonstrating the features of *meta*-materials was synthesized. The transformation process of as-formed hybrid-type film to quite pure MoS_2_ was monitored herein as a function of the green light irradiation dose and intensity of monochromatic *ns*-laser light using SEM, XRD, Raman, XPS, and ToF SIMS.

## Results and Discussion

### Synthesis of hybrid-type MoS_2_ films

**Figure 1:**
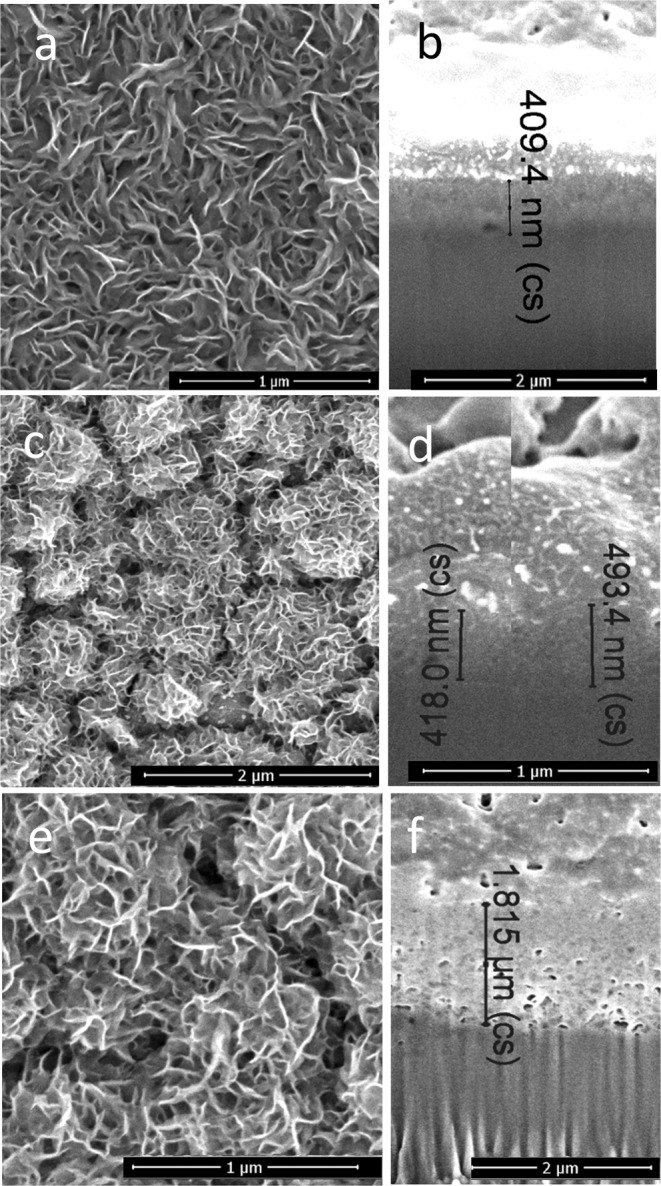
Top-side (**a**) and cross-sectional (**b**) SEM images of nanoplatelet film fabricated on the Ti surface by hydrothermal treatment in the solution containing 5.0 (NH_4_)_6_Mo_7_O_24_ and 90 mmol dm^−3^ thiourea at 220 °C for 10 h. The SEM images of the films fabricated in the same solution with 10 mmol dm^−3^ TEA and with 20 mmol dm^−3^ MEA are depicted in (**c**,**d**) and (**e**,**f**) parts, respectively.

Previously, it has been reported that nanoplatelet-shaped, densely packed, and crystalline MoS_2_ films can be formed directly at the Ti or Mo substrates by hydrothermal processing in the solution containing ammonium heptamolybdate, (NH_4_)_6_Mo_7_O_24_·4H_2_O, and thiourea, CS_2_(NH_2_)_2_, at 220°–225 °C for several hours^30^. In this study, we found that addition of ethanolamines to this synthesis solution results in the formation of significantly thicker (Fig. 1) black-colored film. Besides, the detail SEM inspection of films formed in the triethanolamine (TEA)-containing solution revealed the formation of obviously smaller nanoplatelets and significant fluctuations in the film thickness (Fig. 1d) than in the MEA-containing solutions under the same conditions. Therefore, in the case of MEA addition (Fig. 1e,f), the films formed at a same conditions as in the basic solution are in 3–4 times thicker reaching 2.0 μm, quite uniform in thickness and reproducible.

### Characterization of as-grown products

**Figure 2:**
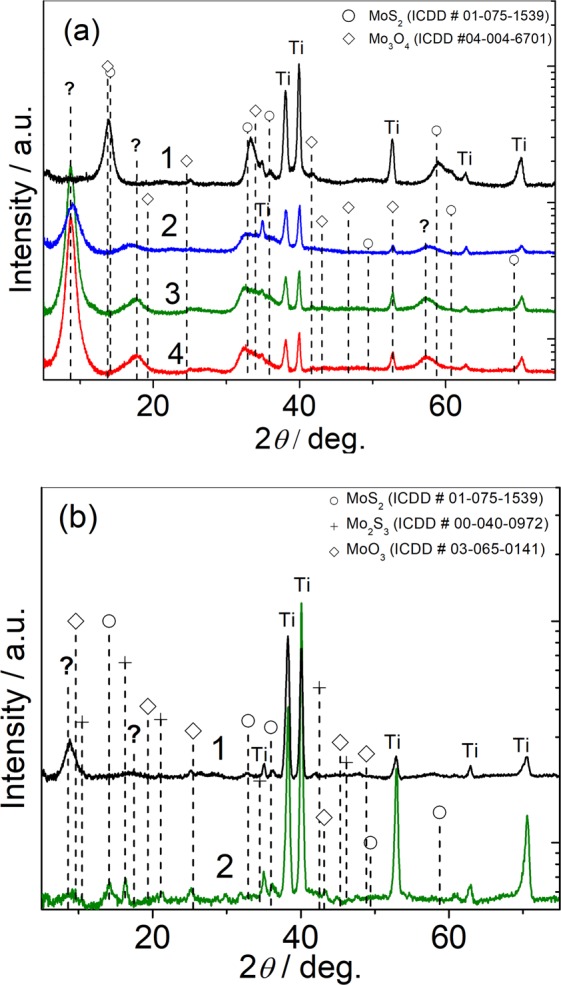
(**a**) XRD patterns of nanoplatelet products formed in the solution containing 5.0 (NH_4_)_6_Mo_7_O_24_ and 90 mmol dm^−3^ thiourea without (1) and with addition of 15 (2), 25 (3) and 35 mmol dm^−3^ (4) MEA by autoclaving at 220 °C for 10 h. (**b**) The same for the nanoplatelet film formed onto the Ti substrate in the solution containing 5.0 (NH_4_)_6_Mo_7_O_24_, 90 thiourea, and 50 mmol dm^−3^ MEA at 220 °C for 5 h before (1) and after (2) green light laser illumination using 8 mJ cm^−2^ dose.

According to glancing angle XRD patterns presented in Fig. 2a, the composition and crystallinity of the fabricated films varied with the concentration of ethanolamine. Comparing to MoS_2_ PDF card no 01-075-1539, quite pure MoS_2_ nanoplatelet species are formed in the basic [5 (NH_4_)_6_Mo_7_O_24_ + 90 mmol L^−1^ CS_2_(NH_2_)_2_] solution. The addition of MEA to this synthesis solution results in the formation of films comprised of significantly lower content of MoS_2_ showing diffraction peaks just from the (101) and (100) planes, and an unknown crystalline material with the main diffraction peaks centered at 2 *theta* angles 8.64°, 17.78°, and 56.24°. Increase in the concentration of MEA results in the formation of the films with larger content of unknown crystallites. From the previous report^31^, hydrothermal processing of solution containing thiourea, (NH_4_)_2_Mo_7_O_24_, and ammonia at 220 °C resulted in the formation of lamellar structure MoS_2_ species in which adjacent layers are filled and expanded by ammonium ions. As has been also reported, for this reason, the intercalation of NH_4_^+^ ions induced the splitting of the main (100%) XRD peak from (002) lattice at 2*Θ* = 14.4° into two located at 9.2° and 18.4° due to the expansion of MoS_2_ interlayers^32^. Therefore, it is reasonable to suspect that in our case the unknown XRD peaks seen at 2*Θ* 8.64° and 17.78° are due to the splitting of the main XRD peak from (002) plane should be seen at the 2*Θ* = 14.1247° angle (PDF Card no. 01-075-1539). We suspect that intercalation of MEA species also results on the splitting of the diffraction peak characteristic for MoS_2_ plane (110) at 2*Θ* = 58.76° into the two ones seen at 2*Θ* = 56.24° and 62.02° (for 50 mmol L^−1^ MEA-containing solution film). At a same time, it seems likely that the content of molybdenum oxide impurities seen in the basic solution film remained quite similar or even decreased in the MEA-containing solution films.

**Figure 3:**
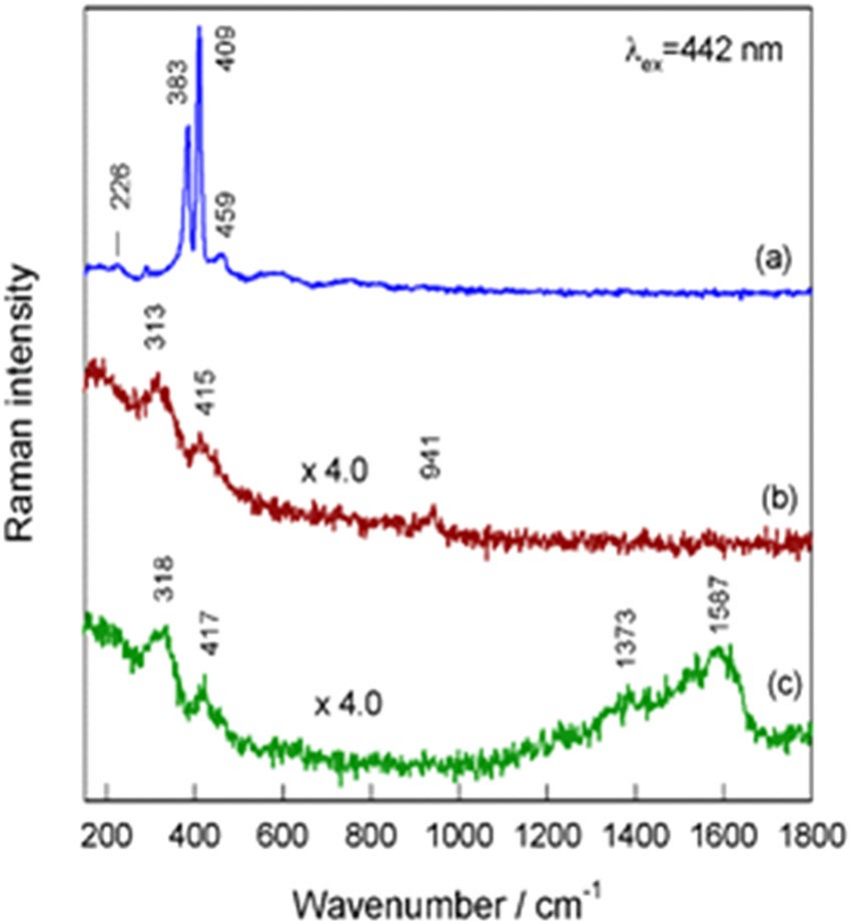
Raman spectra of nanoplatelet film fabricated onto the Ti substrate by hydrothermal treatment at 220 °C for 10 h in the solution of 5.0 (NH_4_)_6_Mo_7_O_24_ and 90 mmol dm^−3^ thiourea without (**a**) and containing 25 (**b**) or 125 mmol dm^−3^ (**c**) MEA. λ_ex_ = 442 nm, cw-laser light power 0.3 mW.

**Figure 4:**
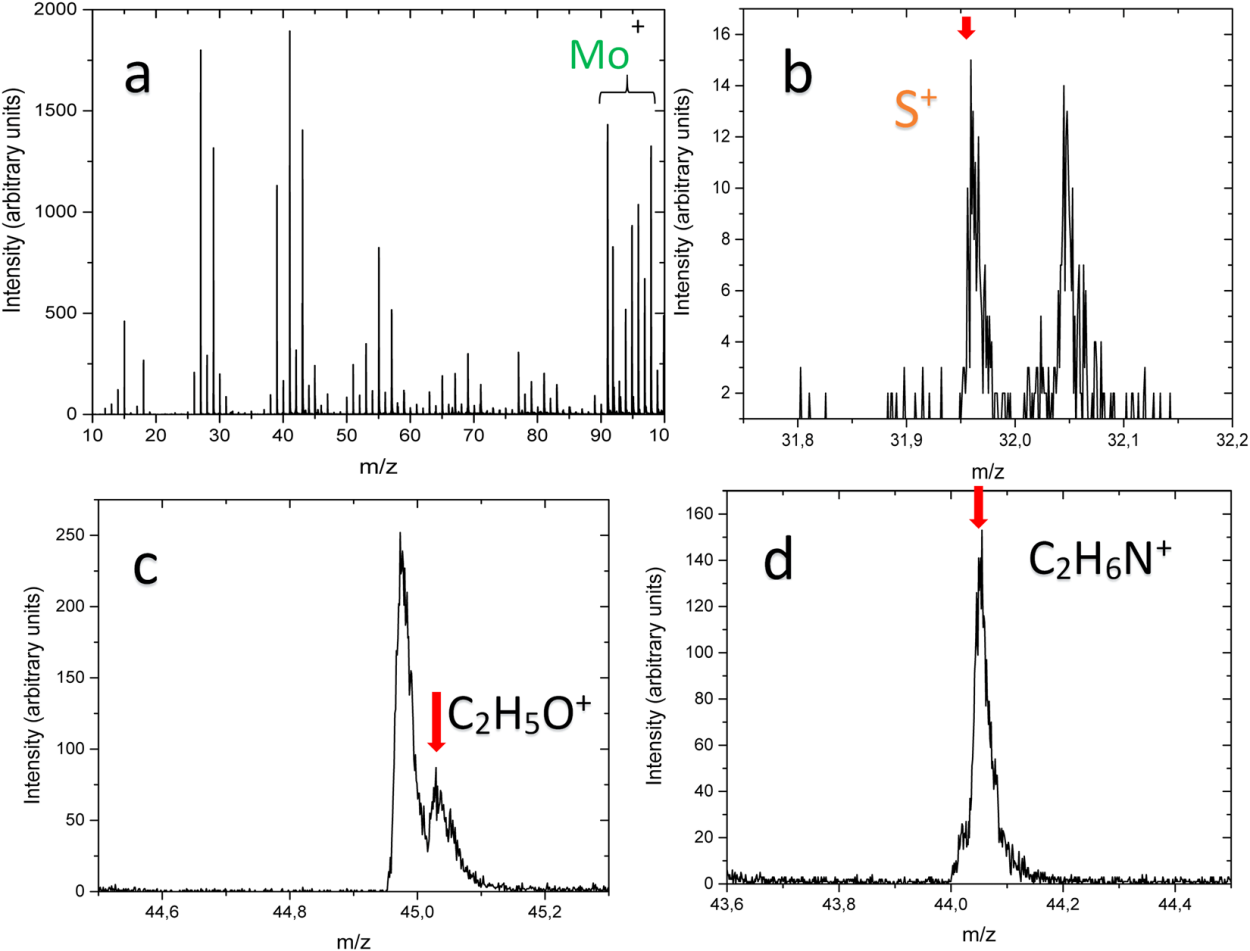
ToF-SIMS spectra of MoS_2_-MEA film fabricated onto the Ti substrate by hydrothermal treatment in the solution containing 5.0 (NH_4_)_2_Mo_6_O_24_, 90 thiourea and 20 mmol dm^−3^ MEA at 220 °C for 10 h.

**Figure 5:**
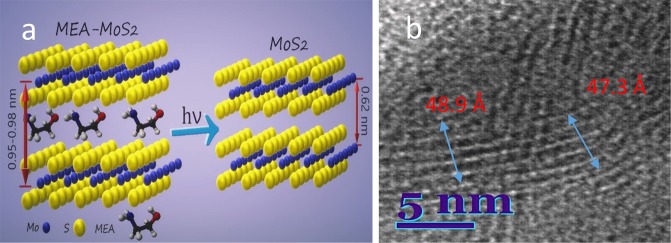
The scheme demonstrating the arrangement of Mo and S atoms in the MEA intercalated MoS_2_ neighborhood nanoplatelets before and after green light laser pulse illumination (**a**). In (**b**), a HRTEM image of MEA-MoS_2_

The typical Raman spectra of nanoplatelet films formed by hydrothermal way in the basic solution and containing MEA are shown in Fig. 3. The doublet at 409 and 383 cm^−1^ is clearly resolved only in the Raman spectrum of basic solution film (Fig. 3a) corresponding well to characteristic vibrational modes of the crystalline MoS_2_ compound^33^. The addition of MEA resulted in the formation of films excelling in poor MoS_2_ purity because of the significant drop of the main Raman modes signals, blue shift of the *E*_2g_ Raman phonon mode of the 2H-MoS_2_ from 382–383 cm^−1^ to 313–318 cm^−1^ and the red-shift of A_1g_ mode position from characteristic 408 cm^−1^ to 415–417 cm^−1^. In addition, the broad bands at 1587 cm^−1^ and 1373 cm^−1^ indicated most likely the presence of –CH_2_- of ethanolamine fragments entrapped in the nanoplatelet material during film formation in the MEA-containing solution. With increasing concentration of MEA in the synthesis reactor, this Raman shoulder strengthened and broadened indicating on the significant MEA contribution to the film composition. It is worth noticing that entrap of –CH_2_- is in accord with our ^1^H and ^13^C NMR spectroscopy investigations revealing the corresponding peak signals at 1.606 and 7.140 ppm in the nanoplatelets formed in the basic solution with 30 mmol dm^−3^ MEA at 225 °C for 10 h. However, while the presence of –CH_2_- groups is clear established, none signal of the ^14^N was identified in the NMR spectrum of as-grown nanoplatelet species from MEA-containing solution perhaps due to a low amount and entrap deeper. Therefore, we have further performed the ToF-SIMS investigations. Static ToF-SIMS spectra taken from the surface of nanoplatelet film grown in the solution containing 30 mmol dm^−3^ MEA are shown in Fig. 4. Firstly, among the overall positive spectra (from m/z = 10 to 100) (Fig. 4a), we spot the occurrence of the several isotopes of molybdenum (92Mo^+^, 94Mo^+^, 95Mo^+^, 96Mo^+^, 97Mo^+^, Mo^+^, 100Mo^+^), whose theoretical distribution is displayed in^31^. Secondly, the region around m/z = 32 (see image in Fig. 4b) indicated the occurrence of the S^+^ ion, along the one of Mo^+^ isotopes, demonstrating the MoS_2_ composition at the surface side whereas the region around m/z = 44 (see image in Fig. 4d) highlighted the presence of C_2_H_6_N^+^ fragment. This one is characteristic of NC_2_H_4_O as well as of NH_2_C_2_H_4_O. It is noteworthy that the discrimination between NC_2_H_4_O and NH_2_C_2_H_4_O is impossible, because the ion beam bombardment of the surface induces the emitted fragments which generally grab H atoms as well as release H atoms. This general phenomenon is uncontrolled and unpredictable, and must be taken into account for interpretation of ToF-SIMS data. From the region around m/z = 45 (see image c in Fig. 4), the fragment C_2_H_5_O^+^ is highlighted and this one reveals NC_2_H_4_O as well as NH_2_C_2_H_4_O. Hence, it can be concluded that the MoS_2_ surface is functionalized by either NC_2_H_4_O or NH_2_C_2_H_4_O molecule fragment. There remains to know whether NC_2_H_4_O/NH_2_C_2_H_4_O molecule binds to the MoS_2_ surface: through the nitrogen atom, or through the oxygen atom. For answering this question, the region around m/z = 106 highlighted the fragment 92MoN^+^, this one does not feature isobaric interference with any other fragments, and can be considered as specific of molybdenum nitride fragment. These data indicate that the NC_2_H_4_O or NH_2_C_2_H_4_O molecule binds to the MoS_2_ surface through the nitrogen atom. Besides, it seems likely than ethanolamine molecule fragments intercalate between the neighbored –S-Mo-S- sheets. This effect was verified by high-resolution transmission microscopy (TEM) observations of as-grown films in the MEA-containing solutions showing the increase of the distance between the Mo atoms in the (002) plane from typical^20^ 0.612 nm to 0.95–98 nm, as shown in Fig. 5.

**Figure 6:**
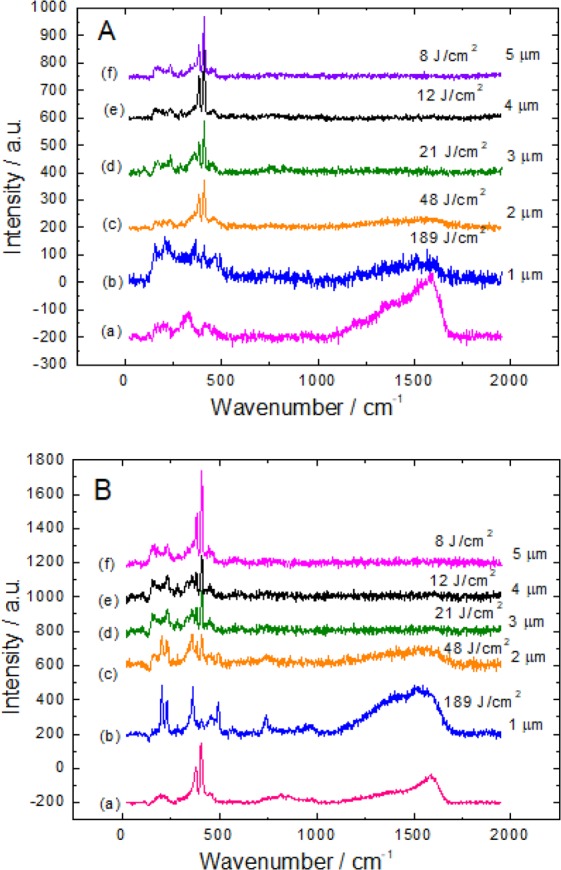
Raman spectra of the film fabricated by hydrothermal treatment of the Ti sample in the solution containing 90 thiourea, 5.0 (NH_4_)_6_Mo_7_O_24_ and 125 mmol dm^−3^ MEA at pH_0_ 10.0 (**A**) and 5.0 (**B**) at 220 °C for 10 h before (a) and after (b–f) green light *ns*-laser illumination at the indicated irradiation dose (average laser power 100 mW; pulse repetition rate 100 kHz). On the right side, the light beam move steps are indicated.

It is also obvious from the Raman spectra (a) presented in Fig. 6 parts A and B that incorporation of ethanolamine fragments into the synthesized nanoplatelets depends on the pH of solution applied. Significantly purer MoS_2_ films with a lower content of MEA were obtained from the slightly acidic solution. In contrast, the Raman modes seen in the frequency region from 120 to 500 cm^−1^ implied the formation in the alkaline solution of the film composed more of molybdenum oxides than MoS_2_ (Fig. 6A–a) whereas MoS_2_ seems dominant in the product synthesized from the same solution kept at a pH of 5.0 (Fig. 6B–a).

Most interesting result of this study was obtained by investigating the effect of green light ps-pulse laser illumination on the composition of this material. The laser-induced treatment resulted in two effects, the first one was the fade of a deep-black color to dark grey and the second one was the formation of significantly pure MoS_2_ nanoplatelets. Figure 6 depicts two sets of Raman spectra taken from the surface of a same nanoplatelet film as-grown in the MEA-containing solution kept at the initial pH 10.0 (A panel) and pH 5.0 (B panel) after green light *ns-*laser illumination with different pulse energies. Surprisingly, within a certain range of light power illumination, their Raman spectra changes drastically: the wide shoulder attributable to organic fragment disappeared whereas the main A_1g_ and E_2g_ modes characteristic to MoS_2_ get strengthened obviously implying on the formation of significantly pure, crystalline MoS_2_. Judging from the A_1g_ and E_2g_ modes shape and positions, the most significant compositional perturbation effect of these films occur at 8 J cm^−2^ fluency. Besides, the electrical conductivity of as-grown MoS_2_ film which equaled to about 4 $\ni$ 10^−3^ S cm^−2^ was found becoming twice higher after green light illumination at 8 J cm^−2^ fluency. Our recent investigations revealed that in the case of high purity few-layered MoS_2_ film, formed by the thermal Mo surface sulfurization, no changes in its composition and structure were viewed even at high ns-pulse lightening fluencies. In the case of MoS_2_ films formed hydrothermally in the ethanolamine-free solution, the damage threshold of the as-grown films was observed at higher light excitation intensities comparing to films grown in the solutions containing ethanolamine.

**Figure 7:**
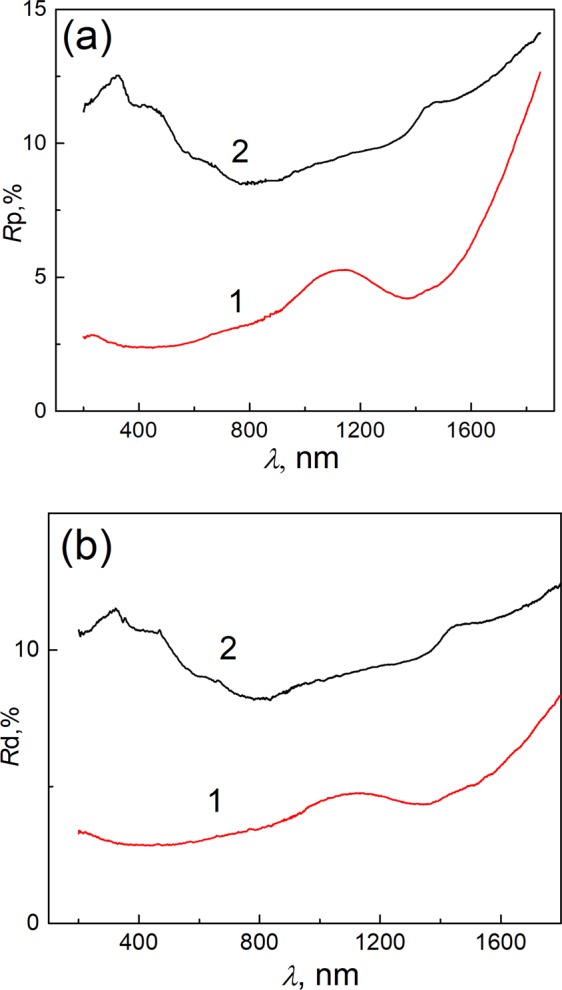
Reflection (**a**) and diffuse-reflection (**b**) spectra of the Ti samples covered with MoS_2_-MEA nanoplatelet film before (1) and after (2) pulse light illumination with 8.0 mJ/cm^2^ irradiation dose.

The optical properties of the MoS_2_-MEA nanoplatelet films before and following pulse light illumination with optimal for laser writing 8.0 mJ cm^−2^ dose were further studied by using UV-Vis- near IR reflection spectroscopy within 200 to 1800 nm wavenumbers range. It is worth noticing that as-grown films are deep-black colored and absorb from 98.5 to 96% of light within all UV-vis region (Fig. 7). Just one reflection shoulder in a vicinity of from about 1000 to 1300 nm peaked at around 1140 nm is characteristic for as-grown films both for reflection and diffuse reflection spectra. However this shoulder is absent in the *R*p(*R*d) *vs λ* spectra of pure MoS_2_ film and should be assigned to the inclusions of organic fragment. We found herein that illumination by pulse green light with 8.0 mJ cm^−2^ dose results in the disappearance of this reflection shoulder well correlating with the Raman spectra variables. It is also obvious from the Fig. 6 that the pulse light illumination resulted in the increase of light reflection within all tested light range. Besides, three new reflection peaks appeared at around 320, 450, and 1480 nm. Based on the Raman spectra the appearance of reflection shoulders in the visible light range after lightening we linked with the formation of purer 2H-MoS_2_ polymorph.

Note that incorporation of metallic 1T-MoS_2_ phase as well MEA fragments can significantly tune the intrinsic conductivity of nanoplatelet film and induce the metallic properties to Mo atoms with octahedral prismatic to Mo atoms with octahedral prismatic coordination^34^.

Variables of the main two vibration modes of the MoS_2_ Raman spectra on the green light irradiation dose for the samples fabricated by hydrothermal synthesis in the MEA-containing solutions at the pH = 10 and pH = 5.0 are shown in the A and B parts of Fig. 6, respectively. As seen, the maximal intensity values of A_1g_ and E^1^_2g_ modes tend to increase for the both specimens with *ns*-laser irradiation dose increase within the tested range up to 20 J cm^−2^. This effect seems not related with the decrease of the MoS_2_ nanoplatelet thickness since in the case of MoS_2_ sheets thickening; A_1g_ peak position usually decreases whereas E^1^_2g_ increases. Quantitative investigations of the intensities ratio of A_1g_ and E^1^_2g_modes, *I*(A_1g_)/*I*((E^1^_2g_), have showed that for both specimens and irradiation wavelengths this ratio tend also to increase with irradiation dose. This subtle effect probably can be explained by light-induced sulfurization of Mo in the oxygen vacancy sites creating new M-S bonds in the defected lattice places.

**Figure 8:**
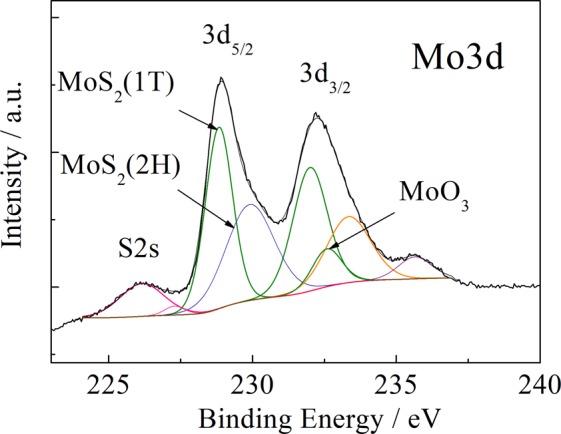
Deconvoluted core level spectra of Mo3d showing the BE of molybdenum in the nanoplatelet film formed at the Ti substrate by hydrothermal treatment in the solution containing 90 thiourea, 5.0 (NH_4_)_6_Mo_7_O_24_), and 45 mmol dm^−3^ MEA at 225 °C for 10 h.

It is commonly accepted that trigonal prismatic arrangements of Mo atoms in the 2H- and 3R-MoS_2_ phases induce them semiconducting properties^24^, whereas the Mo atoms with octahedral prismatic coordination in the 1T-MoS_2_ provides them metallic features^25^. Our X-ray photoelectron spectroscopy investigations revealed that MoS_2_ nanoplatelets fabricated *via* hydrothermal approach in the MEA-containing solutions are composed of the both 2H- and 1T-MoS_2_ fractions, MoO_3_ and S^0^ (Fig. 8). From the recent view point into the nanophotonic objects, the reconfigurable phenomena in the metamaterials are based on the collective coupling of the both resonant plasmonic (metallic) and resonant dielectric parts^35^ aroused upon the pulse laser illuminations. Therefore, co-existence of metallic and semiconducting honeycombed arrangements of molybdenum and sulfur atoms in the MoS_2_ species intercalated with the NC_2_H_4_O^+^ fragments seems allowing the significant compositional changes in this new metamaterial upon excitation with the strong field of the green light laser pulses determined in this study.

**Figure 9:**
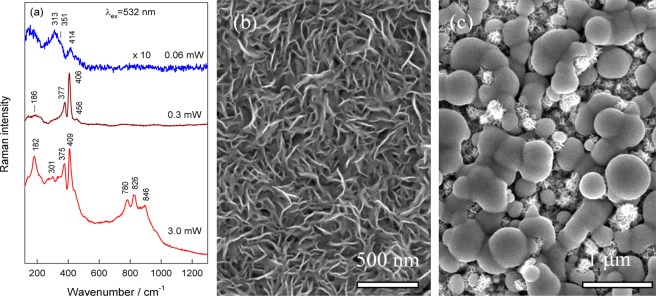
Raman spectra taken at the indicated power (**a**) and the top-side SEM images (**b**,**c**) of the MoS_2_ film formed in the solution and autoclaving conditions as in Fig. 1a after green light ns-laser illumination with 0.3 (**b**) and 3.0 mW (**c**) power energy.

Judging from the green light illumination intensity-dependent Raman spectra (Fig. 9a), high-intensity illumination with a green light ns-laser irradiation dose resulted in the photo-degradation of 2H-MoS_2_ and 1T-MoS_2_ phases and formation of molybdenum oxides. The formation of Mo oxides was confirmed by appearance of clearly resolved new Raman peaks at 780, 826 and 846 cm^−1^ (Fig. 9a, 3 mW spectrum) ascribed to oxides in^36^. The reshaping of the nanoplatelets structure was clearly observed and verified by SEM (Fig. 9c). In contrast, when the optimal lightening regime was applied, the composite material transforms to obviously purer material with prevailed 2H MoS_2_ phase without structure changes (Fig. 9a, 0.3 mW spectrum).

## Conclusions

In this paper, we report about the one-pot hydrothermal synthesis of hybrid-type MoS_2_ nanoplatelet shaped films intercalated with ethanolamine fragments as a new metamaterial for *ps*-laser green light writing. The intercalation of ethanolamine molecule fragments between adjacent MoS_2_ layers was verified by HRTEM observations revealing increase in the distance between the Mo atoms, from the typical 0.62 to 0.95–0.98 nm, and the splitting of XRD peak of the (002) plane, typical for 2H-MoS_2_ crystalline phase at the 2*Θ* = 14.125° angle, into two peaks, namely at the 2*Θ* = 8.64° and 17.78°. The green light *ps*-laser illumination with a suitable fluency was found resulting in the fade of the deep-black color of the film, the detachment of attached NC_2_H_4_O^+^ species from the Mo atoms, and transformation of hybrid MoS_2_ material into significantly pure and less resistant MoS_2_. These findings were verified by ToF-SIMS, Raman and light reflection spectroscopy investigations of as-grown nanoplatelet films after green light *ps*-laser pulse illumination with optimized 8.0 mJ cm^−2^ irradiation dose. From the electrical conductivity measurements, green light ns-laser illumination at the irradiation dose of 8 J/m^2^ results in our MoS_2_-based film resistance twice decrease and black color fade.

We demonstrated that nanolayered composites constructed from 2H-MoS_2_ (semiconducting) and 1T-MoS_2_ (metallic) components and entrapped ethanolamine fragments can be ascribed to saturable absorbers. Compared to MoS_2_/graphene-based metamaterials light-mater interaction in the coupled 1T-MoS_2_/2H-MoS_2_ atomically thin layers proceeds under visible light ps-laser irradiation. Therefore, it seems reasonable to suspect the promising utilizations of these films as non-reversible photo-conducting material for ns-laser writing and information storage.

## Methods

### Materials and chemicals

Ammonium heptamolybdate tetrahydrate (NH_4_)_6_Mo_7_O_24_ · 4H_2_O (99.5%) was obtained from Reachem (Slovakia), thiourea, (NH_2_)_2_CS (99%) and ethanolamines were purchased from Sigma, Germany and used as received. Aqueous solutions were prepared using deionized water (18.4 MΩ). Ti foil (99.7%) 0.127 mm thick was purchased from Aldrich.

### Synthesis

Pure MoS_2_ nanoflowered species were synthesized as follows. At first, 15 mL of aqueous solution containing 5.0 ammonium heptamolybdate and 90 mmol dm^−3^ thiourea was prepared and poured in a Teflon line stainless steel autoclave in volume of 25 mL. The synthesis was conducted at 220°–225 °C for 5 to 10 h using 10 °C min^−1^ ramp. The synthesized products were collected by centrifugation, rinsed carefully with pure water and dried. To obtain densely packed MoS_2_ nanoplatelets film, the Ti specimen (12 × 12 mm) was inserted inside the reactor. Obtained products after collection were rinsed and dried in air. For hybridization of MoS_2_ nanoplatelets, monoethanolamine (MEA) or triethanolamine (TEA) in concentration from 10 to 125 mmol L^−1^ was added to the synthesis reactor.

### Laser-induced selective phase transition procedure

The LISPTP procedure was performed in order to investigate the influence of green (*λ* = 532 nm) ns laser illumination on the structural and compositional transformations of hybrid-type MoS_2_-based nanoplatelet films. The laser irradiations were performed in air, perpendicular to the film surface, using the central part of the laser beam, with a diameter of 1 mm having a rather homogeneous intensity. Experimental setup consisted of ns-laser Baltic HP (irradiation wavelength - 532 nm, pulse duration - 10 ns, pulse repetition rate - 100 kHz) beam expander and galvoscanner with f-*theta* lens focusing objective (focal distance −80 mm for 532 nm), similar to previous reported by us^26^. Areas of 1 × 1 mm^2^ were scanned in line stacks. Distances between adjacent lines and scanning speed (100–500 mm/s) were adjusted to ensure same pulse overlap in both directions. Distance between adjacent laser pulses (*d*) was varied between 1 and 5 μm. Average laser power was varied between 1 and 20 mW. Diameter of the focused laser beam was 26 μm.

### Raman

The 532 nm excited Raman spectra were recorded using inVia (Renishaw) spectrometer equipped with thermoelectrically cooled (−70 °C) CCD camera, 1800 lines/mm grating, and a microscope. The 532 nm beam of the CW solid state laser was used as an excitation source. The position of Raman spectra bands on the wavenumber axis was calibrated by the silicon peak at 520.7 nm. The 50x/0.75 NA objective was used during the measurements. The beam was focused to a 2 μm diameter spot size on the sample surface. The integration time was 100 s. Spectra are not smoothed or averaged.

### ToF SIMS

Time-of-flight secondary ions mass spectrometry (TOF SIMS) analysis of the atomic and molecular secondary ions that are emitted from the tested solid state surface of the nanoplatelet film when bombarded with ions were conducted with a ToF SIMS IV instrument from ION-ToF GmbH. An Ar + 10 keV ion beam was used as analysis beam at a current of 0.6 pA. Four different locations on the surface were accounted for the ToF SIMS analyses, in order to ensure the reproducibility and significance of the results.

### SEM and XRD

The morphology and elemental composition of the products obtained was investigated using a scanning electron microscope (FEI Quatra 200F) and a Cross Beam Workstation Auriga equipped with a field emission gun and EDX spectrometer. The glancing angle XRD spectra were collected on a D8 diffractometer (Bruker AXS, Germany), equipped with a Göbel mirror as a primary beam monochromator for CuK_α_ radiation.

X-rays photoelectron spectra (XPS) were collected using the ESCALAB MKII spectrometer equipped with the new XR4 twin anode. The non-monochromatised MgK_α_ X-ray source was operated at *hν* = 1253.6 eV with the 300 W power (20 mA/15 kV) and the pressure in the analysis chamber was lower than 5 × 10^−7^ Pa during spectral acquisition. The spectra were acquired with the electron analyzer pass energy of 20 eV for narrow scans and the resolution of 0.05 eV and with a pass energy of 100 eV for survey spectra. All spectra were recorded at the 90° take-off angle and calibrated using the C 1 s peak at 284.6 eV. The spectra calibration, processing and fitting routines were done using the Avantage software (5.918) provided by Thermo VG Scientific. Core level peaks of Mo3d were analyzed using a nonlinear Shirley-type background.

NMR experiments were carried out on the Bruker AVANCE IIIHD spectrometer operating at 9.4 T magnetic field. Background suppression pulse sequence was used to obtain 1H spectrum of 200 ppm width. Spectrum of the 31 k points was recorded with 64 number of scans using 2.5 μs and 98 W pulses. For additional decoupling, magic angle spinning technique was used at a rate of 10 kHz with 4 mm zirconia rotor. Powdered adamant was measured as well for external reference.

The reflectance spectra of the samples were recorded in the wavelength rage of 200–1700 nm using the Shimazu UV-Vis-NIR spectrometer equipped with the MPS-3100 integrating sphere. The specular reflectance of the light from the film surface was calculated using an optical model of two layers.
